# *Lactobacillus rhamnosus* MY-1 alleviates deoxynivalenol-induced oxidative stress, inflammation, and gut microbiota dysbiosis both *in vivo* and *in vitro*

**DOI:** 10.3389/fmicb.2026.1750402

**Published:** 2026-02-16

**Authors:** Yukai Lin, Ruibiao Wang, Jie Yao, Hanqing Zhao, Zuhua Yu, Bo Wen, Yanyan Jia, Chengshui Liao, Rongxian Guo, Lei Wang, Xiaojing Xia, Yanzhao Xu, Ke Ding

**Affiliations:** 1 College of Animal Science and Veterinary Medicine, Henan Institute of Science and Technology, Xinxiang, China; 2 Laboratory of Functional Microbiology and Animal Health, Henan University of Science and Technology, Luoyang, China; 3 Luoyang Key Laboratory of Live Carrier Biomaterial and Animal Disease Prevention and Control, Henan University of Science and Technology, Luoyang, China; 4 The Key Lab of Animal Disease and Public Health, Henan University of Science and Technology, Luoyang, China

**Keywords:** deoxynivalenol, gut microbiota, inflammation, *Lactobacillus rhamnosus* MY-1, oxidative stress

## Abstract

**Introduction:**

Deoxynivalenol (DON), a prevalent mycotoxin in grains and feed, poses a serious threat to animal health by inducing intestinal dysfunction. While some probiotics are known to mitigate DON toxicity, the multifaceted protective effects of *Lactobacillus rhamnosus* MY-1—a strain with high DON-degradation capacity and a proven safety profile—require comprehensive evaluation. This study aimed to systematically assess the ability of MY-1 to alleviate DON-induced oxidative stress, inflammation, and gut microbiota dysbiosis, and to elucidate its underlying mechanisms.

**Methods:**

Investigations were conducted *in vitro* using IPEC-J2 cells and *in vivo* using a BALB/c mouse model. We examined the effects of MY-1 on cell viability, ultrastructure, oxidative stress markers (MDA, T-AOC), inflammatory cytokines (TNF-α, IL-1α, IL-4), apoptosis-related genes (BAX, Caspase-3, BCL-2), and tight junction protein (ZO-1, Occludin, Claudin-1) expression. Gut microbiota composition was analyzed via alpha (Chao1, Simpson, Shannon) and beta diversity indices.

**Results:**

The MY-1 supernatant restored IPEC-J2 cell viability and ameliorated DON-induced ultrastructural damage. MY-1 alleviated oxidative stress by reducing MDA and enhancing T-AOC, while inhibiting pro-inflammatory cytokines (TNF-α, IL-1α) and promoting the anti-inflammatory cytokine IL-4. In mice, MY-1 mitigated DON-induced growth inhibition and intestinal pathological damage, restored tight junction protein expression, and regulated apoptosis-related genes. Gut microbiota analysis showed that MY-1 reversed DON-induced dysbiosis, restoring alpha diversity and beta diversity structure, and modulated the abundances of dominant genera such as *Bacteroides* and *Dubosiella*.

**Discussion:**

This study demonstrates that *Lactobacillus rhamnosus* MY-1 exerts comprehensive protective effects against DON-induced intestinal toxicity through integrated mechanisms, including direct detoxification, antioxidant and anti-inflammatory activities, and microbiota modulation. These findings underscore the value of MY-1 as a well-characterized probiotic candidate for mitigating mycotoxin effects in animal production.

## 1 Introduction

Deoxynivalenol (DON), a type B trichothecene mycotoxin produced primarily by *Fusarium graminearum* and *Fusarium oxysporum*, widely contaminates grains such as wheat, corn, and barley, posing a serious threat to both food security and livestock production (Robinson et al., 2023). Owing to its stable physicochemical properties, DON is difficult to effectively remove through conventional processing and readily enters the feed chain, where it can accumulate in livestock and poultry (Lauren and Smith, 2001). Chronic DON intake in animals can induce various toxic effects, including anorexia, growth retardation, immunosuppression, and intestinal dysfunction, leading to a 15–30% reduction in feed conversion rate and thereby causing substantial economic losses to the animal husbandry industry (Reddy et al., 2018; Serviento et al., 2018).

The primary mechanisms by which deoxynivalenol impairs animal health involve the induction of oxidative stress, inflammatory responses, and disruption of the gut microbiota (Zeng et al., 2025). At the cellular level, DON inhibits protein synthesis and induces severe oxidative stress, resulting in substantial accumulation of reactive oxygen species (ROS) and weakening the cellular antioxidant defense capacity (Kouadio et al., 2005; Garofalo et al., 2025). Furthermore, DON can trigger intestinal inflammation (Hu et al., 2024). It also significantly impairs the intestinal barrier through downregulation of tight junction proteins (including Occludin, Claudin-1, Claudin-3, and ZO-1) and promotes cell apoptosis while disrupting the cytoskeletal organization critical for maintaining tight junctions, ultimately compromising the intestinal epithelial barrier (Zhang et al., 2022; Miao et al., 2024). Additionally, DON exposure markedly reshapes the gut microbiota, reducing the abundance of beneficial bacteria such as *Faecalibacterium prausnitzii* and *Mucispirillum schaedleri*. This dysbiosis interacts with disturbances in the gut environment, collectively exacerbating intestinal damage (Jin et al., 2023). Impairment of intestinal barrier function not only hinders nutrient absorption but may also allow bacterial and endotoxin translocation, leading to systemic inflammation and seriously compromising animal production performance and immune function (Matar et al., 2024). Therefore, developing effective strategies to mitigate DON-induced intestinal toxicity has become an important focus of current research.

Currently, detoxification strategies for DON primarily encompass physical adsorption, chemical degradation, and biotransformation. However, the application of physical and chemical methods is often limited by their moderate efficiency, potential safety risks, or adverse effects on the nutritional quality of feed. Consequently, the development of safe and efficient biological detoxification approaches has become a major research focus (Ji et al., 2016; Guo et al., 2020). Previous studies have demonstrated that certain bacterial strains, including *Bacillus licheniformis* YB9, *Bacillus subtilis* ASAG216, and *Lactobacillus plantarum* JM113, can alleviate DON-induced toxicity in animal models through mechanisms such as mitigating growth inhibition, oxidative stress, gut microbiota dysbiosis, and enhancing intestinal barrier function (Wu et al., 2018; Wang et al., 2020; Jia et al., 2021). These findings highlight the potential of probiotics in counteracting mycotoxin-induced intestinal injury.

Lactic acid bacteria (LAB), recognized as safe probiotics, exhibit favorable gastrointestinal tolerance and intestinal colonization capacity, and can mitigate mycotoxin toxicity through adsorption, biotransformation, antioxidant activity, and immune regulation (Guan et al., 2023; Krishnan et al., 2023). The strain investigated in this study, *Lactobacillus rhamnosus* MY-1, was previously isolated and characterized in our laboratory. It possesses a comprehensively assessed safety profile, a fully sequenced genome, and demonstrates a high *in vitro* DON degradation rate (93.34% within 48 h) involving both adsorption and enzymatic mechanisms (Yao et al., 2024). To provide a more comprehensive evaluation of this strain’s potential, we employed integrated in vitro (IPEC-J2 cells) and *in vivo* (BALB/c mouse) models to systematically investigate the protective effects of *L. rhamnosus* MY-1 against DON-induced oxidative stress, inflammatory responses, intestinal barrier damage, and gut microbiota dysbiosis. This study therefore seeks to characterize *L. rhamnosus* MY-1 as another well-defined strain within this field, providing comprehensive in vitro and in vivo efficacy data to expand the scientific knowledge base on probiotics with DON-mitigating potential.

## 2 Materials and methods

### 2.1 Reagents, strains, cells, animals

DON standard was purchased from Qingdao Prubang Bioengineering Co., Ltd. Assay kits for superoxide dismutase (SOD), glutathione peroxidase (GSH-Px), MDA, and T-AOC were obtained from Nanjing Jiancheng Technology Co., Ltd. AG RNAex Pro RNA extraction reagent was sourced from Hunan Aikerui Bioengineering Co., Ltd. Hoechst 33342 staining solution, Annexin V-PE apoptosis detection kit, and CCK-8 reagent were supplied by Shanghai Biyuntian Biotechnology Co., Ltd. 2 × RealStar Fast dye qPCR premix and StarScript II genomic DNA removal reverse transcription premix reagents were purchased from Beijing Kangrun Chengye Biotechnology Co., Ltd. All qPCR primers were synthesized by Biotechnology (Shanghai) Co., Ltd. DMEM cell culture medium was obtained from Situofan Biotechnology Co., Ltd., and fetal bovine serum was procured from Shanghai Jiase Biotechnology Co., Ltd. Additional reagents including 0.25% trypsin solution and penicillin–streptomycin dual antibiotic solution were supplied by Wuhan Saiweier Biotechnology Co., Ltd.

The strain *Lactobacillus rhamnosus* MY-1 was isolated and preserved in our laboratory. The porcine intestinal epithelial cell line IPEC-J2 was also maintained in our laboratory. A total of 48 six-week-old male BALB/c mice with an initial body weight of 16 ± 2 g were purchased from the Animal Center of Zhengzhou University for use in the experiments.

### 2.2 Preparation of culture supernatant and degradation supernatant from *Lactobacillus rhamnosus* MY-1

*Lactobacillus rhamnosus* MY-1 stored at −80 °C was inoculated into MRS liquid medium for activation. A 1% (v/v) inoculum was then transferred into fresh MRS medium and incubated anaerobically at 37 °C for 24 h. The bacterial culture was collected and centrifuged at 12,000 r/min for 5 min, after which the supernatant was filtered through a 0.22 μm membrane to obtain the cell-free supernatant (CFS). The CFS was aliquoted and stored at −20 °C until use. For preparation of the degradation supernatant, MY-1 culture grown for 24 h was co-incubated with DON for 48 h. The mixture was then centrifuged and filtered following the same procedure, and the resulting supernatant was collected as the degradation supernatant and stored at −20 °C.

### 2.3 Culture and treatment of IPEC-J2 cells

Cells were cultured in DMEM complete medium supplemented with 10% fetal bovine serum and 1% penicillin–streptomycin in a 37 °C incubator with 5% CO₂. When cells reached 70–80% confluence, they were digested and passaged using 0.25% trypsin. The cell suspension was adjusted to a density of 1 × 10^5^ cells/mL and seeded into 96-well plates (100 μL per well) or 6-well plates (1 mL per well). After 4 h of incubation to allow for full adhesion, the cells were used for treatments. The DON challenge concentration (0.25 μg/mL) and the MY-1 CFS intervention dose (5%, v/v) were selected based on preliminary dose–response experiments, which were conducted to optimize experimental conditions for subsequent mechanistic evaluation. The cells were then divided into four treatment groups: the control group received only complete culture medium; the MY-1 group was treated with 5% (v/v) of *L. rhamnosus* MY-1 CFS; the DON group was exposed to 0.25 μg/mL of DON toxin; and the MY-1 + DON group was treated with 5% (v/v) of the degradation supernatant obtained by co-culturing MY-1 with DON. All treatments were applied continuously for 36 h, after which cells were harvested for subsequent analysis.

### 2.4 Cell viability and morphological observation

Cell viability was assessed using the CCK-8 assay. After treatment, 10 μL of CCK-8 solution was added to each well, and the plates were further incubated for 1 h in the incubator. The absorbance of each well was then measured at a wavelength of 450 nm using a microplate reader. Cell viability was expressed as a percentage relative to the control group.

For observation of cellular morphology, samples were sequentially fixed with 2.5% glutaraldehyde (in 0.1 M PBS buffer) and 1% osmium tetroxide, thoroughly rinsed with PBS, and dehydrated using a graded acetone series (30 to 100%). Following dehydration, the samples were infiltrated stepwise with a 1:1 mixture of acetone and Epon-812 embedding resin, followed by pure resin, and then embedded and polymerized at 60 °C. The polymerized blocks were sectioned into 60–80 nm ultrathin slices using an ultramicrotome. After double-staining with uranyl acetate and lead citrate, the sections were observed and imaged under a transmission electron microscope at 20,000 × magnification. All cell experiments were performed in three independent replicates (*n* = 3).

### 2.5 Determination of cellular antioxidant indices

Levels of MDA, SOD activity, and T-AOC were detected using commercially available assay kits (Jiancheng Bioengineering Institute, China), in accordance with the procedures described in a previous study (Tian et al., 2021). Each assay was conducted with three independent biological replicates (*n* = 3).

### 2.6 Detection of apoptosis

Cell apoptosis was detected using an Annexin V-PE apoptosis detection kit with *in situ* fluorescence microscopy. The specific procedure was as follows: after treatment under respective conditions, the culture medium from IPEC-J2 cells was removed, and 0.1 mL of fixative solution was added followed by incubation at room temperature for 10 min. The fixative was then discarded, and cells were washed twice with PBS (3 min per wash), with residual liquid completely aspirated. Next, 0.5 mL of Hoechst 33258 staining solution was added and allowed to stain for 5 min. After removal of the staining solution, cells were rinsed 2–3 times with PBS. Subsequently, 195 μL of Annexin V-PE binding buffer was added to resuspend the cells, followed by addition of 5 μL of Annexin V-PE and gentle mixing. The samples were incubated at room temperature (20–25 °C) in the dark for 10–20 min. Apoptosis was assessed by observing red fluorescence under a fluorescence microscope. Apoptosis experiments were performed in three independent replicates (*n* = 3).

### 2.7 Animal experiment design and sample collection

All animal experiments were approved by the Institutional Animal Ethics and Welfare Committee. Prior to the experiment, the animals were acclimatized for one week under standard conditions (12-h light/dark cycle, with free access to food and water). To minimize bias, a completely randomized design was employed for group assignment. Specifically, each mouse was assigned a unique identification number, and group allocation (Control, MY-1, DON, MY-1 + DON) was determined using a computer-generated random number sequence. The mice were randomly assigned to four groups (*n* = 12) and treated as follows: the control group received 0.2 mL of sterile PBS daily by oral gavage; the MY-1 group was administered 0.2 mL of *L. rhamnosus* MY-1 bacterial suspension (1 × 10^9^ CFU/mL) daily; the DON group was fed a diet containing 3 mg/kg DON; and the MY-1 + DON group received both the 3 mg/kg DON-containing diet and daily gavage of an equivalent dose of MY-1 bacterial suspension. In this study, a DON dose of 3 mg/kg diet was selected, which corresponds to an estimated daily intake of approximately 0.45 mg DON/kg body weight (assuming a daily feed intake of 3 g and an average mouse body weight of 20 g). This dose is relevant to field exposure scenarios where contaminated feed may contain DON levels ranging from 1 to 5 mg/kg, thereby providing a realistic model for evaluating probiotic intervention under practical conditions. Wherever feasible, blinding was implemented during the experiment and outcome assessment. The personnel responsible for daily gavage, body weight/feed intake recording, and tissue collection were unaware of the group allocations (single-blind). Additionally, histopathological evaluation and quantitative PCR data analysis were performed by investigators blinded to the treatment groups. The experiment lasted 42 days. Body weight and feed intake were recorded weekly. At the end of the experiment, blood was collected from the orbital venous plexus, and serum was separated by centrifugation and stored at −20 °C. The mice were then euthanized by cervical dislocation under anesthesia, and segments of the duodenum, jejunum, ileum, and cecum were rapidly dissected. A portion of intestinal tissue was fixed in 4% paraformaldehyde for histological analysis, and the remainder was snap-frozen at −80 °C for subsequent molecular and microbiological assays. For all subsequent analyses, six mice per group (*n* = 6) were randomly selected.

### 2.8 Histopathological analysis

Fixed mouse organ tissue samples stored at 4 °C were embedded in paraffin, and sections were prepared from all organs. These sections were stained with hematoxylin and eosin (H&E) and examined under a microscope. Similarly, fixed samples from different intestinal segments were embedded, sectioned, and H&E-stained for microscopic observation. Villus height (VH) and crypt depth (CD) were measured using ImageJ software, and the villus-to-crypt ratio (V/C) was calculated.

### 2.9 Quantitative real-time PCR

Frozen mouse organ tissues stored at −80 °C were homogenized, and total RNA was extracted using the AG SteadyPure Universal RNA Extraction Kit. RNA concentration and A260/A280 ratio were measured with a spectrophotometer. The extracted RNA was then reverse-transcribed into cDNA using the AG StarScript II Genomic DNA Removal Reverse Transcription Premix. Relative expression levels of target genes were determined by quantitative real-time PCR (qRT-PCR) using ChamQ Universal SYBR qPCR Master Mix (Novozymes, China) on a Roche LightCycler 480 system. With glyceraldehyde-3-phosphate dehydrogenase (GAPDH) as the housekeeping gene, relative gene expression was calculated using the 2-ΔΔCt method. Primer sequences for both cell and mouse samples are listed in Supplementary Table 1.

### 2.10 Gut microbiome sequencing and analysis

Following DNA extraction from cecal contents and quality verification via Nanodrop and agarose gel electrophoresis, the V3–V4 hypervariable regions of the bacterial 16S rRNA gene were amplified using barcoded primers 338F (5′-ACTCCTACGGGAGGCAGCA-3′) and 806R (5′-GGACTACHVGGGTWTCTAAT-3′). The resulting amplicons were purified, quantified, pooled in equimolar ratios, and used to construct sequencing libraries with the Illumina TruSeq Nano DNA LT Library Prep Kit. High-throughput paired-end sequencing was performed on the Illumina NovaSeq 6,000 platform. After demultiplexing based on barcode sequences, raw reads were processed using the QIIME2 pipeline, where denoising and amplicon sequence variant (ASV) clustering were conducted with DADA2. Taxonomic assignment was performed against the SILVA 138 database. All samples were rarefied to an even sequencing depth prior to downstream analysis. Alpha diversity was assessed using the Chao1, Shannon, and Simpson indices, and beta diversity was evaluated by Bray–Curtis distance and visualized via principal coordinates analysis (PCoA). Microbial composition was summarized at the phylum and genus levels. Differentially abundant taxa between groups were identified using linear discriminant analysis effect size (LEfSe) with a Kruskal–Wallis test (*p* < 0.05) for significance and a linear discriminant analysis (LDA) score threshold > 4.0; results were presented as LDA score histograms and cladograms to illustrate phylogenetic discriminative features.

### 2.11 Statistical analysis

All experimental data were analyzed using IBM SPSS Statistics 25 software. Normality was assessed by the Shapiro–Wilk test and homogeneity of variances by Levene’s test. Data meeting both assumptions were analyzed by one-way ANOVA followed by Tukey’s HSD test. For data violating the normality assumption (e.g., certain microbiota and gene expression data), appropriate transformations (log10, square-root, or arcsine-square-root) were applied. If transformed data still deviated from normality, non-parametric tests were used: the Kruskal–Wallis test followed by the Mann–Whitney U test with Bonferroni correction for pairwise comparisons. Microbiome compositional data were analyzed within QIIME2 using appropriate transformations (e.g., centered log-ratio). Differential abundance was assessed by LEfSe with the non-parametric Kruskal–Wallis test (*p* < 0.05) and LDA score > 4.0. Bar graphs were generated using GraphPad Prism 8. Data are presented as mean ± standard deviation (SD). Differences were considered statistically significant at *p* < 0.05 and highly significant at *p* < 0.01. The ‘n’ values represent independent biological replicates (animals or independent experiments).

## 3 Results

### 3.1 *Lactobacillus rhamnosus* MY-1 alleviates DON-induced cytotoxicity in IPEC-J2 cells *in vitro*

**Figure 1:**
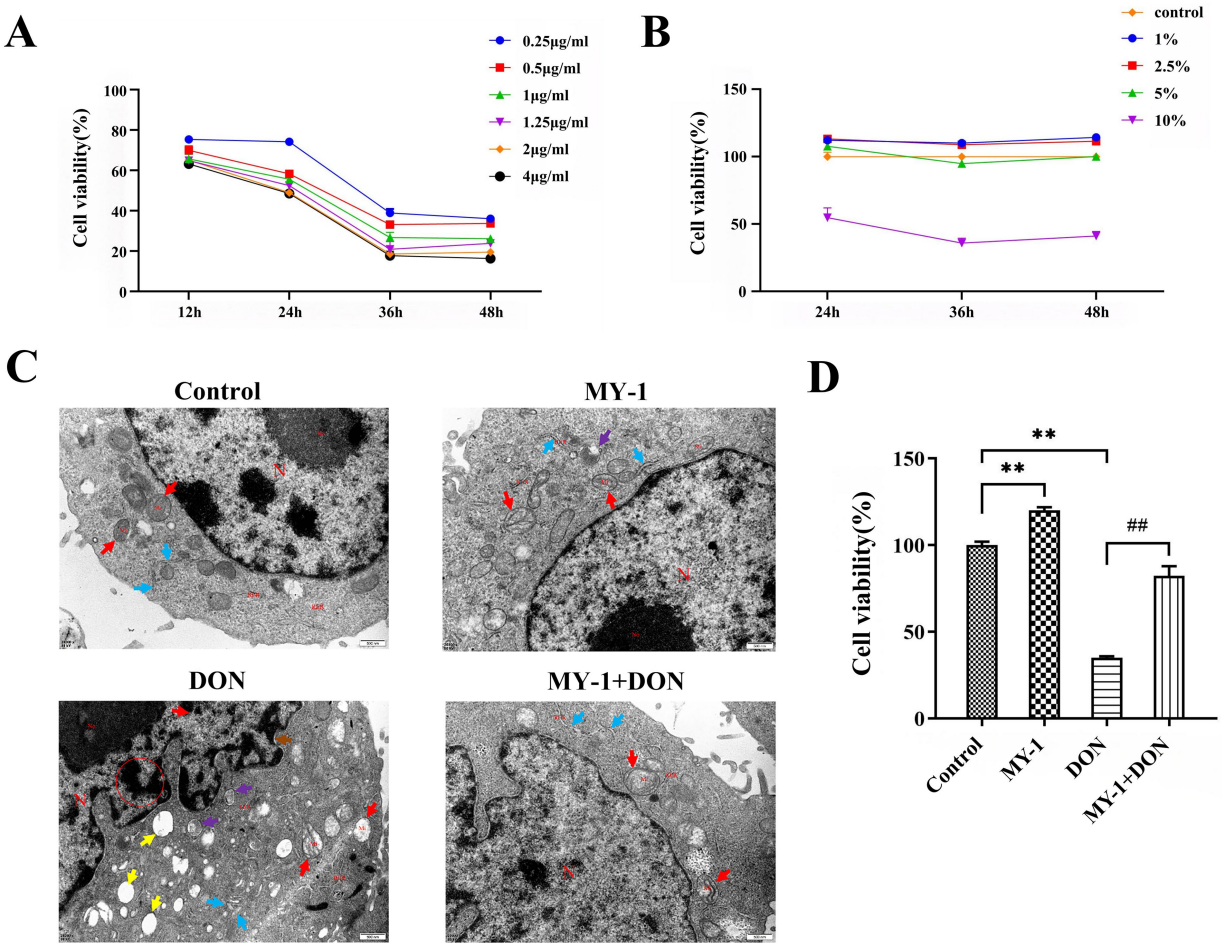
*Lactobacillus rhamnosus* MY-1 alleviates DON-induced cytotoxicity in IPEC-J2 cells. **(A)** Effect of different DON concentrations on cell viability. **(B)** Effect of *L. rhamnosus* MY-1 CFS on cell viability. **(C)** Representative transmission electron micrographs of cell morphology. **(D)** Cell viability following treatment with the degradation supernatant. The data is expressed as mean ± standard deviation (mean ± SD). Compared with the control group, *p < 0.05 and **p < 0.01 indicate statistical significance; Compared with the DON treatment group, #*p* < 0.05 and ##*p* < 0.01 indicate statistical significance.

To evaluate the protective effect of *Lactobacillus rhamnosus* MY-1 against DON-induced injury, we first assessed the viability and morphology of IPEC-J2 cells. DON exposure significantly reduced cell viability in a time- and concentration-dependent manner. Treatment with 0.25 μg/mL DON for 36 h decreased cell viability to approximately 50% (Figure 1A). A 5% concentration of *L. rhamnosus* MY-1 CFS maintained 100% cell viability over 48 h, whereas higher concentrations led to reduced viability (Figure 1B). Based on these pilot experiments, 0.25 μg/mL DON and 5% CFS were chosen as the optimal challenge and intervention doses, respectively, for all subsequent mechanistic investigations. Morphological analysis by transmission electron microscopy revealed that DON induced severe cellular damage, including nuclear condensation, mitochondrial swelling, and endoplasmic reticulum dilation. In contrast, the degradation supernatant group exhibited only mild abnormalities, similar to the control and MY-1 supernatant groups (Figure 1C). Moreover, cells treated with the degradation supernatant showed significantly restored viability compared to those treated with DON alone (Figure 1D). These results demonstrate that *L. rhamnosus* MY-1 effectively alleviates DON-induced cytotoxicity.

### 3.2 *Lactobacillus rhamnosus* MY-1 alleviates DON-induced oxidative stress, inflammatory response, and impairment of barrier function *in vitro*

**Figure 2:**
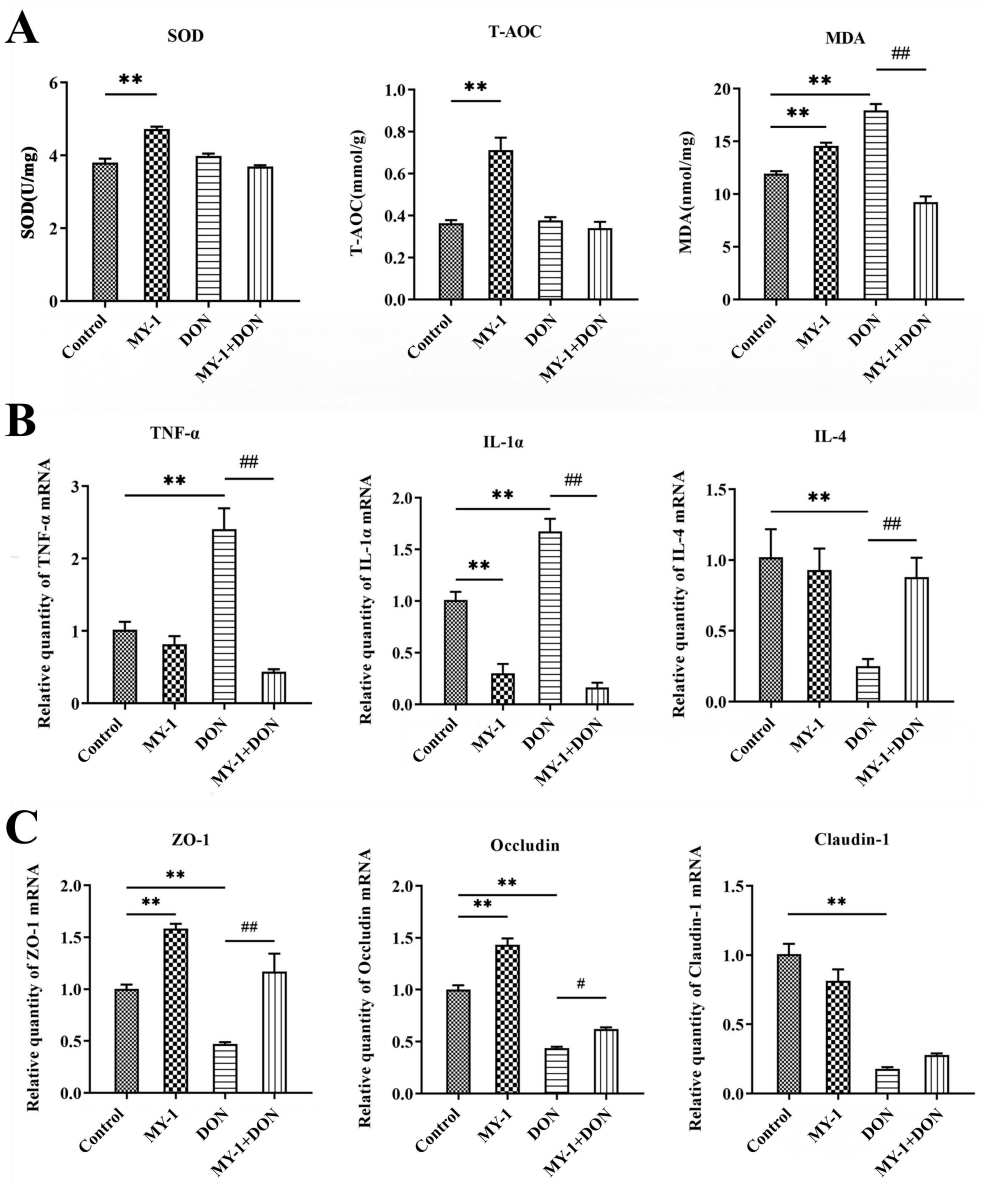
*Lactobacillus rhamnosus* MY-1 alleviates DON-induced oxidative stress, inflammatory response, and intestinal barrier damage in IPEC-J2 cells. Effects of DON, *L. rhamnosus* MY-1 CFS, and degradation supernatant on: **(A)** oxidative stress markers (MDA, T-AOC, SOD); **(B)** relative mRNA expression levels of the pro-inflammatory cytokines TNF-α and IL-1α, and the anti-inflammatory cytokine IL-4; **(C)** relative mRNA expression levels of tight junction proteins ZO-1, Occludin, and Claudin-1. The data is expressed as mean ± standard deviation (mean ± SD). Compared with the control group, *p < 0.05, **p < 0.01; compared with the DON treatment group, #*p* < 0.05, ##*p* < 0.01.

In IPEC-J2 cells, exposure to deoxynivalenol significantly induced oxidative stress, as evidenced by elevated MDA levels, whereas treatment with the CFS of *Lactobacillus rhamnosus* MY-1 alone enhanced antioxidant enzyme activity. Compared with the control group, the DON-treated group showed no significant changes in T-AOC or SOD activity, but exhibited a marked increase in MDA. In contrast, treatment with the MY-1 degradation supernatant significantly reduced MDA content and improved overall antioxidant capacity (Figure 2A). At the mRNA level, DON strongly induced an inflammatory response, significantly upregulating TNF-*α* and IL-1α while downregulating IL-4. The degradation supernatant notably reversed these changes, reducing pro-inflammatory cytokines and restoring expression of the anti-inflammatory cytokine IL-4 (Figure 2B). Furthermore, DON significantly downregulated the expression of the tight junction proteins ZO-1, Occludin, and Claudin-1, thereby compromising intestinal barrier integrity. Treatment with the CFS of *L. rhamnosus* MY-1 alone upregulated ZO-1 and Occludin expression, while the degradation supernatant significantly restored the expression levels of both proteins (Figure 2C). Collectively, these results demonstrate that *L. rhamnosus* MY-1 alleviates DON-induced oxidative stress, inflammatory response, and impairment of barrier function in IPEC-J2 cells.

### 3.3 Effect of *Lactobacillus rhamnosus* MY-1 on DON-induced apoptosis in IPEC-J2 cells

**Figure 3:**
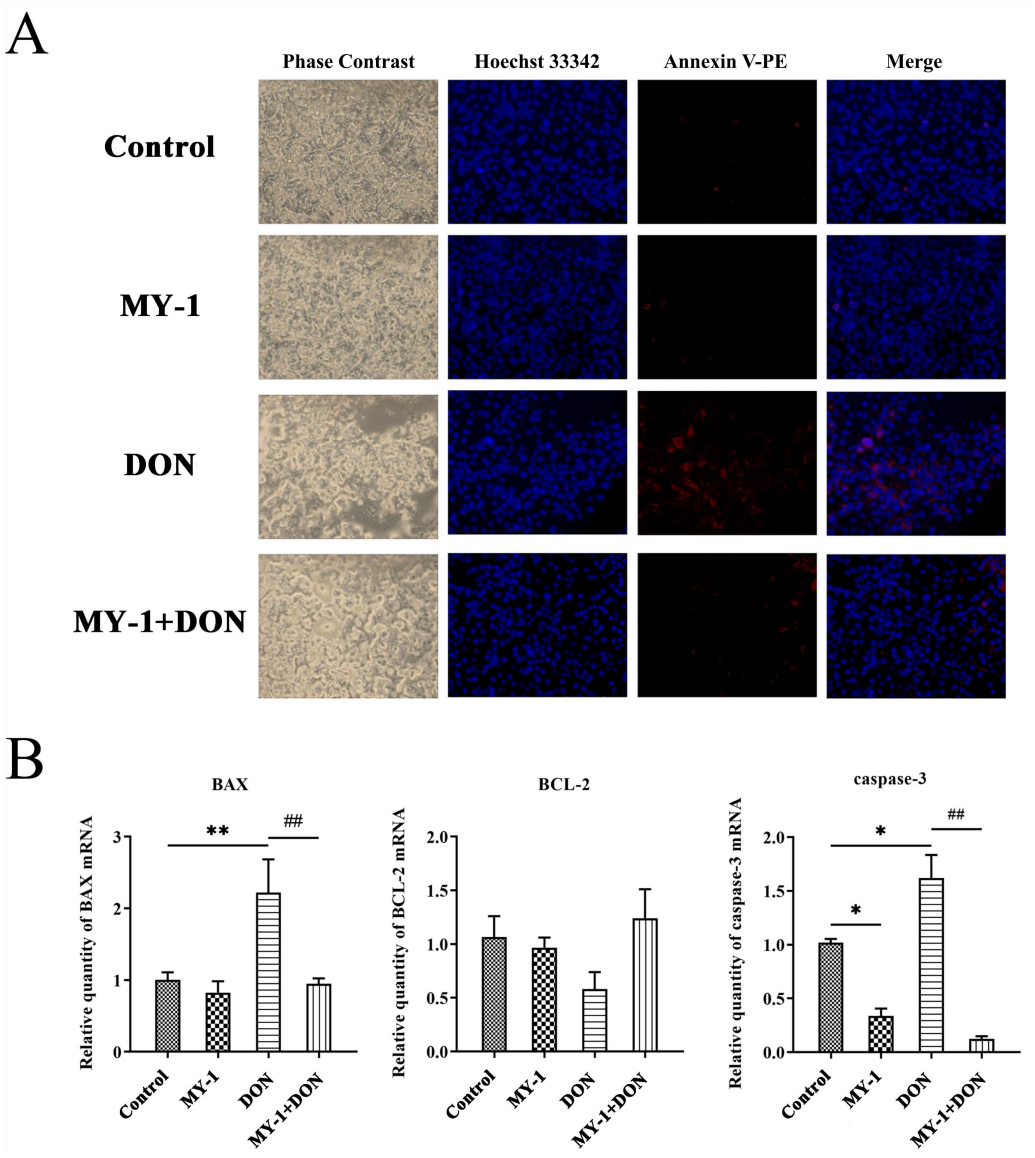
*Lactobacillus rhamnosus* MY-1 alleviates deoxynivalenol-induced apoptosis in IPEC-J2 cells. **(A)** Representative fluorescence micrographs of apoptotic cells. **(B)** Relative mRNA expression levels of apoptosis-related genes *BAX*, *BCL-2*, and *Caspase-3*. The data is expressed as mean ± standard deviation (mean ± SD). Compared with the control group, *p < 0.05, **p < 0.01; compared with the DON treatment group, #*p* < 0.05, ##*p* < 0.01.

Fluorescence microscopy analysis (Figure 3A) revealed that IPEC-J2 cells in the control group exhibited blue nuclei with no apparent signs of apoptosis. The supernatant group showed only minimal apoptotic red fluorescence. In contrast, the DON group displayed extensive necrotic and apoptotic features, whereas the degradation supernatant group exhibited markedly reduced apoptosis and improved cellular morphology compared to the DON group. Further analysis of apoptosis-related gene expression (Figure 3B) demonstrated that the mRNA levels of the pro-apoptotic genes BAX and Caspase-3 were upregulated in the DON group. However, the expression of both genes in the degradation supernatant group was lower than that in the DON group, with Caspase-3 expression even lower than that in the control group. These results collectively indicate that DON treatment was associated with upregulation of apoptosis-related genes and corresponding morphological changes, while the degradation supernatant was linked to an attenuation of these effects, suggesting its potential protective role in IPEC-J2 cells.

### 3.4 *Lactobacillus rhamnosus* MY-1 alleviates DON-induced growth impairment and intestinal damage in mice

**Figure 4:**
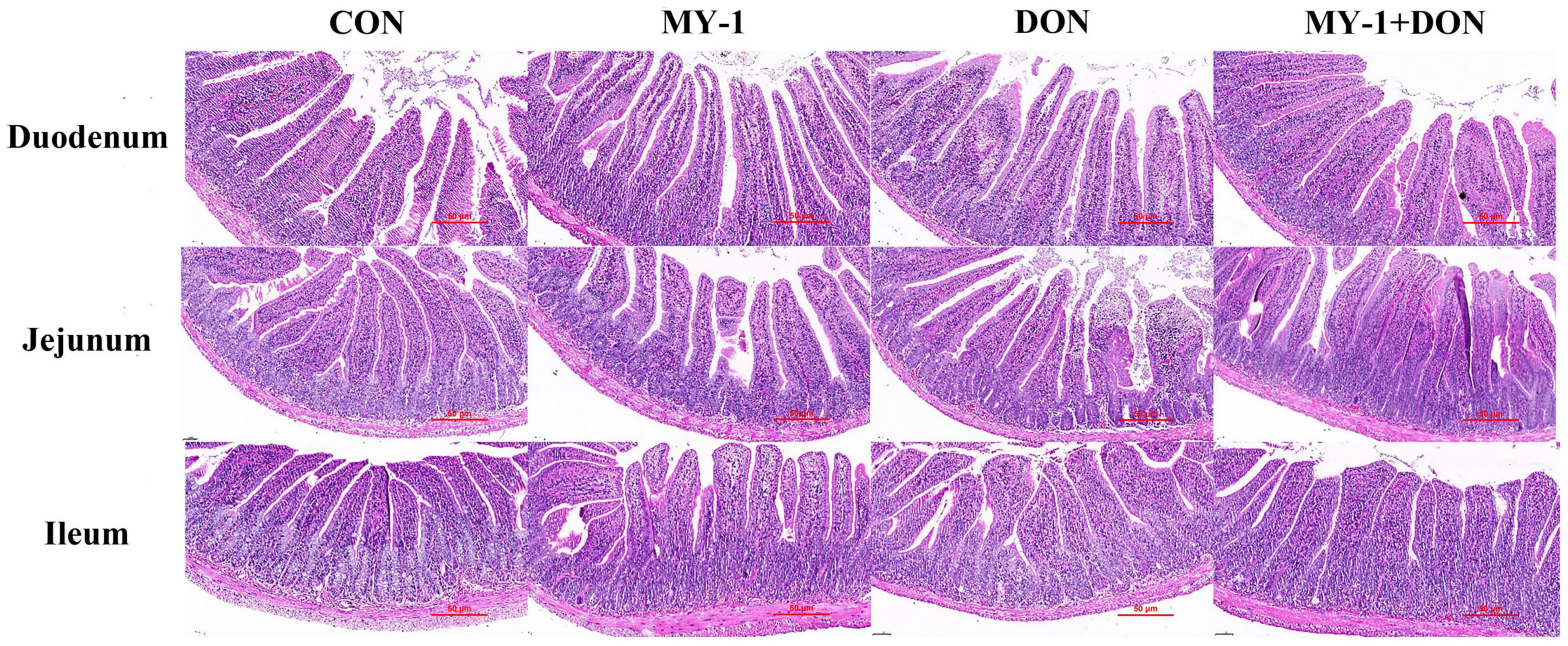
*Lactobacillus rhamnosus* MY-1 alleviates deoxynivalenol-induced intestinal injury in mice. Representative H&E-stained sections of duodenum, jejunum, and ileum.

*In vivo* experiments showed that mice in the control and *L. rhamnosus* MY-1 groups remained healthy throughout the trial with no mortality. In contrast, one mouse in the DON group died on day 28 after exhibiting severe lethargy and anorexia; this animal was excluded from all subsequent analyses. The mortality in the DON group, although of low incidence, is consistent with the systemic toxic effects of DON exposure, likely resulting from severe intestinal barrier disruption, systemic inflammation, or metabolic dysregulation. The remaining mice in the DON group displayed persistent signs of toxicity, including mental exhaustion, reduced activity, and ruffled fur, whereas animals in the control, MY-1, and MY-1 + DON groups remained active and exhibited normal grooming behavior. Although no significant differences in initial body weight or feed intake were observed among the groups, the weight gain rate in the DON group during the 14–42 day challenge period was significantly lower than that of the control group. In contrast, the MY-1 + DON group showed a higher weight gain rate compared to the DON group, suggesting that DON inhibits growth and that MY-1 alleviates this effect (Supplementary Table 2; Supplementary Figure 1). To further investigate the impact of MY-1 on DON-induced intestinal injury, H&E staining was performed on the duodenum, jejunum, and ileum (Figure 4), and villus height, crypt depth, and the villus height-to-crypt depth ratio were measured. Histological analysis revealed intact and well-organized intestinal structures in the control, MY-1, and MY-1 + DON groups, whereas the DON group exhibited significant damage in the jejunal villi. Statistical analysis (Supplementary Table 3) indicated that ileal VH in the MY-1 group was significantly higher than in the control group. In the DON group, VH was significantly reduced in the duodenum and jejunum, CD was significantly increased in the jejunum and ileum, and V/C was significantly elevated in the duodenum and jejunum. By comparison, the MY-1 + DON group showed significantly higher VH and V/C values across all intestinal segments relative to the DON group, with ileal VH even exceeding that of the control group. These findings demonstrate that DON disrupts intestinal barrier integrity, while *L. rhamnosus* MY-1 effectively mitigates this damage and exerts a protective effect on the intestine.

### 3.5 *Lactobacillus rhamnosus* MY-1 restores DON-impaired tight junction and apoptosis-related gene expression *in vivo*

**Figure 5:**
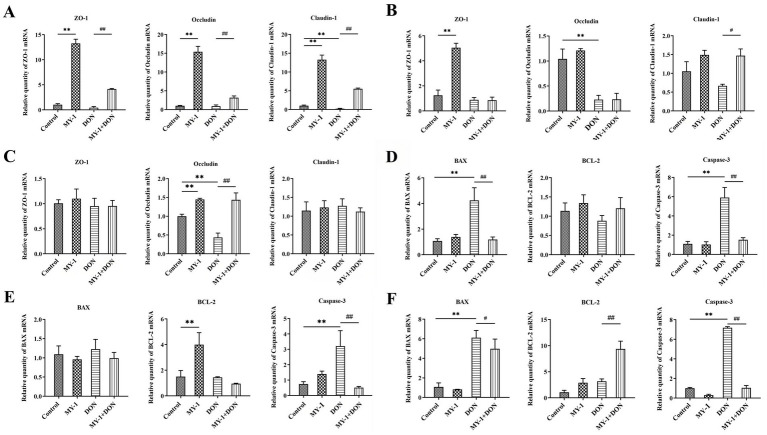
Effects of *Lactobacillus rhamnosus* MY-1 on relative mRNA expression levels of tight junction proteins (ZO-1, occludin, claudin-1) in the **(A)** duodenum, **(B)** jejunum, and **(C)** ileum, and apoptosis-related genes (BAX, BCL-2, caspase-3) in the **(D)** duodenum, **(E)** jejunum, and **(F)** ileum of mice following DON challenge. The data is expressed as mean ± standard deviation (mean ± SD). Compared with the control group, *p < 0.05, **p < 0.01; compared with the DON treatment group, #*p* < 0.05, ##*p* < 0.01.

The mRNA expression levels of tight junction proteins and apoptosis-related genes in the duodenum, jejunum, and ileum of mice were analyzed using quantitative real-time PCR. In the duodenum (Figure 5A), DON exposure significantly downregulated Claudin-1 and moderately reduced ZO-1 and Occludin expression. Administration of *Lactobacillus rhamnosus* MY-1 significantly reversed these changes, increasing the expression of all three tight junction proteins. In the jejunum (Figure 5B), DON markedly suppressed Occludin and reduced Claudin-1 expression, while MY-1 intervention significantly elevated Claudin-1 levels. In ileal tissue (Figure 5C), no significant differences in ZO-1 and Claudin-1 were observed among groups; however, DON significantly decreased Occludin expression, which was restored by MY-1 co-treatment. Regarding apoptosis-related genes, DON significantly upregulated BAX and Caspase-3 expression in the duodenum (Figure 5D) and ileum (Figure 5E), and increased Caspase-3 levels in the jejunum (Figure 5F). MY-1 intervention effectively counteracted these effects, significantly reducing BAX and Caspase-3 expression in the duodenum and ileum, and lowering Caspase-3 in the jejunum. Additionally, MY-1 significantly enhanced BCL-2 expression in the ileum compared to the DON group. These results demonstrate that *Lactobacillus rhamnosus* MY-1 effectively alleviates DON-induced intestinal barrier damage by modulating the expression of tight junction proteins and apoptosis-related genes.

### 3.6 *Lactobacillus rhamnosus* MY-1 modulates gut microbiota diversity in DON-exposed mice

**Figure 6:**
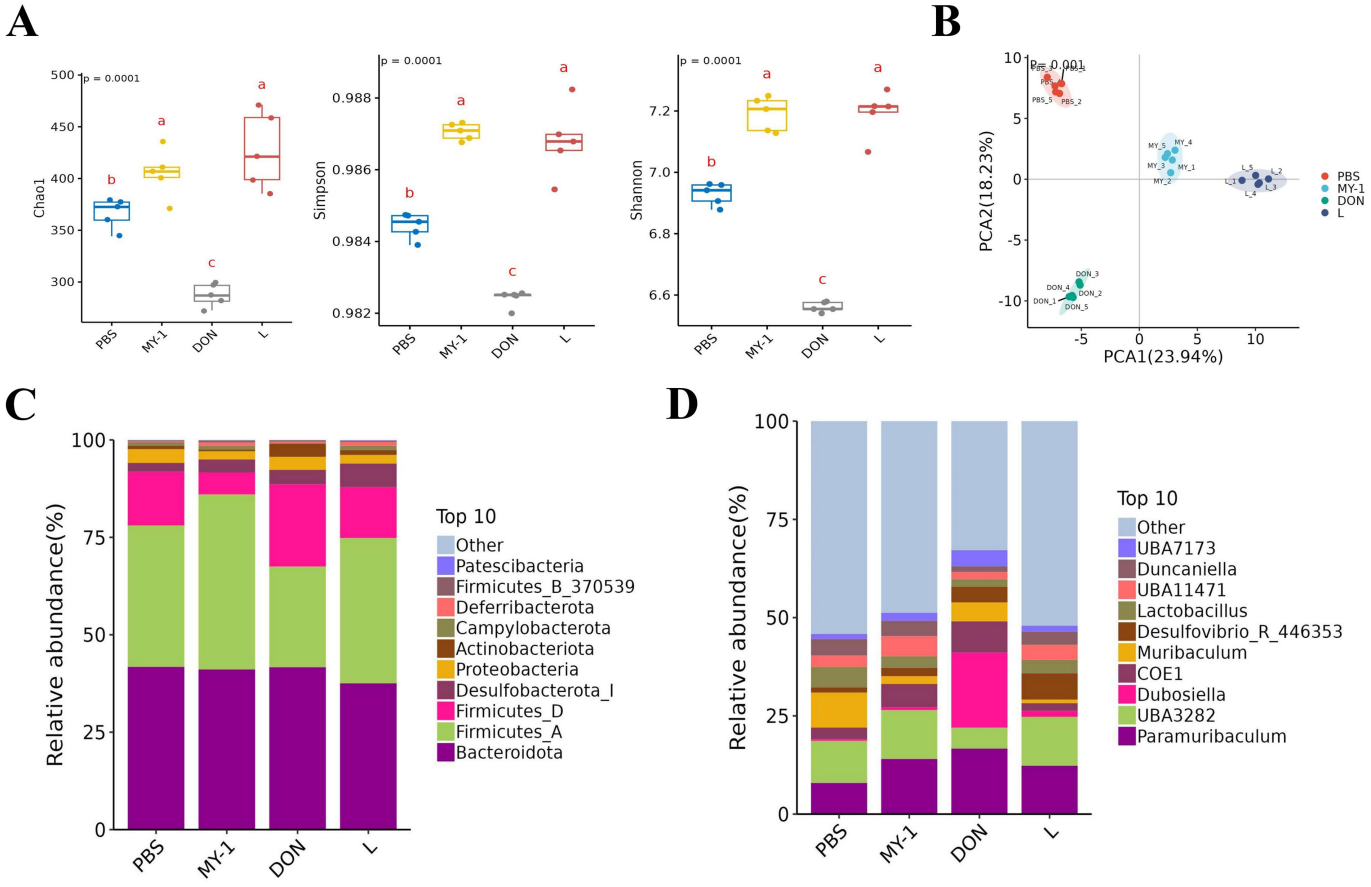
*Lactobacillus rhamnosus* MY-1 regulates gut microbiota diversity in DON-exposed mice. **(A)** Alpha diversity indices, including Chao1, Simpson, and Shannon indices. **(B)** Beta diversity analyzed by PCoA. **(C)** Relative abundance of dominant bacterial phyla. **(D)** Relative abundance of dominant bacterial genera.

Analysis of cecal microbiota alpha diversity revealed that, compared to the control group, the MY-1 group exhibited significant increases in the Chao1, Simpson, and Shannon indices, while the DON group showed significant decreases. All three indices were significantly higher in the MY-1 + DON group than in the DON group (Figure 6A). Principal coordinates analysis (PCoA) of beta diversity indicated good intra-group sample reproducibility and clear separation in microbial composition among the four groups (Figure 6B). At the phylum level, *Bacteroidota*, *Firmicutes*, *Desulfobacterota*, and *Proteobacteria* were the dominant phyla, with no significant differences observed among the four groups (Figure 6C). At the genus level, *Bacteroides* and *Dubosiella* were the predominant genera. The relative abundance of *Bacteroides* and *Dubosiella* was higher in the DON group than in the control group (Figure 6D). These results suggest that *Lactobacillus rhamnosus* MY-1 alleviates DON-induced gut microbiota dysbiosis by modulating its structural composition and diversity.

### 3.7 *Lactobacillus rhamnosus* MY-1 alters the gut microbiota composition in DON-exposed mice

**Figure 7:**
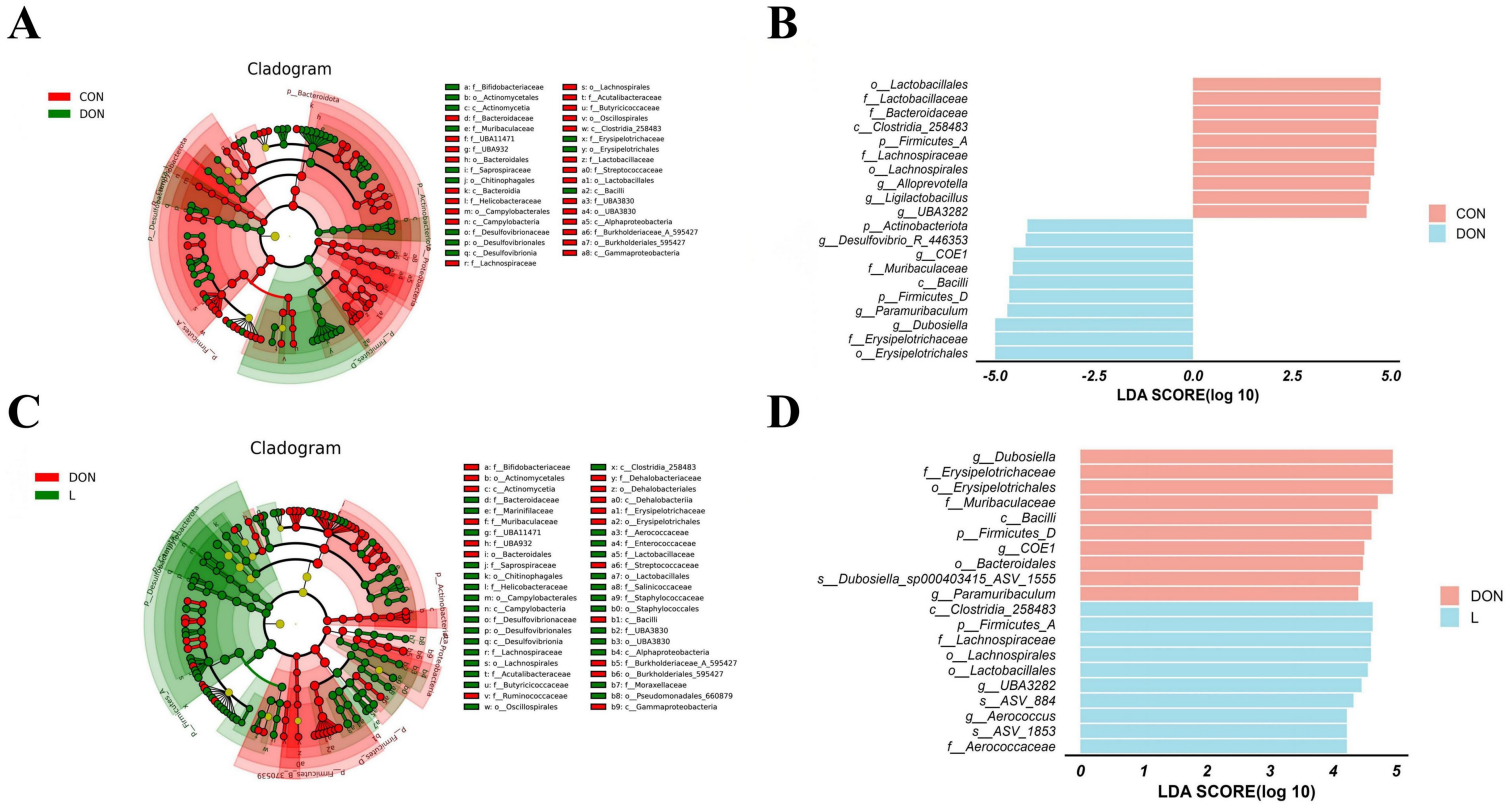
Effect of *Lactobacillus rhamnosus* MY-1 on gut microbiota composition in deoxynivalenol-exposed mice. **(A)** LEfSe analysis cladogram showing differentially abundant bacterial taxa between the DON and control groups. **(B)** LDA scores of taxa significantly enriched in the DON group compared to controls. **(C)** LEfSe cladogram of differential taxa between the MY-1 + DON and DON groups. **(D)** LDA scores of taxa significantly enriched in the MY-1 + DON group compared to the DON group.

LEfSe analysis revealed that, compared with the control group, DON exposure significantly enriched several bacterial taxa in the mouse gut, including *Erysipelotrichales*, *Erysipelotrichaceae*, *Dubosiella*, *Paramuribaculum*, *Firmicutes_D*, *Bacilli*, and *Desulfovibrio_R446353* (Figures 7A,B). Furthermore, supplementation with *Lactobacillus rhamnosus* MY-1 effectively reversed these DON-induced changes and significantly increased the relative abundance of beneficial taxa such as *Clostridia_258483*, *Firmicutes_A*, *Lachnospiraceae*, *Lactobacillales*, and *Aerococcus* (Figures 7C,D). These findings indicate that DON exposure induces gut microbiota dysbiosis, marked by the proliferation of pro-inflammatory and opportunistic pathogenic bacteria. In contrast, *L. rhamnosus* MY-1 counteracts DON-induced dysbiosis by promoting the colonization and growth of beneficial bacteria, thereby restoring a more balanced microbial community.

## 4 Discussion

The widespread contamination of grain and feed with deoxynivalenol induces multi-organ toxicity in animals, causing systemic damage such as immunosuppression, neurotoxicity, and reproductive disorders (Tu et al., 2023). As the primary site of toxin contact after ingestion, the gastrointestinal tract is particularly vulnerable: DON impairs intestinal barrier function, which can lead to systemic inflammation and metabolic dysregulation (Li et al., 2025). Given the limitations of physical and chemical detoxification methods—such as low efficiency and potential negative effects on feed quality—biological detoxification has become a focus of research (Yao and Long, 2020). Several studies have reported the DON-degrading potential of probiotics. For example, *Bacillus subtilis* QR-03 shows a degradation rate exceeding 80%, and *Bacillus velezensis* BL-14 degrades 82.63% of DON at a concentration of 1 μg/mL, highlighting the promise of probiotics in detoxification applications (Lai et al., 2021; Sun et al., 2024). However, certain harmful strains also exhibit high degradation capacity. *Bacillus cereus* JG05, for instance, can degrade more than 80% of DON but is a known foodborne pathogen associated with emetic and diarrheal illnesses (Yu et al., 2016; Dietrich et al., 2021). Similarly, *Pseudomonas aeruginosa* NF011 effectively protects wheat spikes and grains from *Fusarium graminearum* infection by producing phenazine-1-carboxamide (PCN), thereby reducing DON synthesis (Sun et al., 2021). Yet, as an opportunistic pathogen with strong environmental adaptability and multiple virulence factors, *P. aeruginosa* poses serious risks to immunocompromised individuals, such as those with cystic fibrosis (Jurado-Martín et al., 2021). These findings underscore that microbial detoxification capability does not equate to safety. The use of pathogenic or uncharacterized microorganisms in feed additives carries potential risks, emphasizing the importance of screening DON-degrading strains that combine high detoxification efficiency with biological safety and potential probiotic properties.

Lactic acid bacteria (LAB) are generally recognized as safe (GRAS) microorganisms with a long history of use in fermented foods and sustainable agriculture (Raman et al., 2022). Furthermore, LAB contribute to intestinal health and enhance host disease resistance through multiple mechanisms, including the production of antibacterial metabolites, competitive exclusion of pathogens, and modulation of host immunity (Zielińska and Kolożyn-Krajewska, 2018). In this context, the present study highlights the distinctive value of *Lactobacillus rhamnosus* MY-1. Unlike the potentially hazardous degrading strains previously mentioned, *L. rhamnosus* MY-1 is a well-established safe LAB strain that has demonstrated both safety and a high DON degradation rate of 93.34% (Yao et al., 2024). However, most existing studies on LAB-mediated mitigation of DON toxicity have focused either on *in vivo* protective effects or have been limited to a single mechanistic level (de Souza et al., 2020; Maidana et al., 2022). This study integrates the strategies of efficient degradation, cellular protection, and systematic microecological regulation at both cellular and animal levels, comprehensively elucidating the multi-target synergistic mechanism by which *Lactobacillus rhamnosus* MY-1 alleviates deoxynivalenol-induced toxicity.

The intestine, serving as the primary barrier against external stimuli, is a major target organ for DON exposure. Previous studies have confirmed the multiple detrimental effects of this mycotoxin on intestinal health (Wang et al., 2024; Zeng et al., 2025). However, the systematic molecular mechanisms through which probiotics alleviate DON-induced intestinal injury remain incompletely understood. Related studies indicate that DON induces dose-dependent toxicity in IPEC-1 and IPEC-J2 cells *in vitro*, and leads to impaired growth performance and intestinal damage in mice in vivo (Diesing et al., 2011; Ye et al., 2023). In the present study, exposure to 0.25 μg/mL DON in vitro caused significant cytotoxicity in IPEC-J2 cells, markedly reducing cell viability and inducing severe ultrastructural damage, including nuclear condensation and mitochondrial swelling. In the animal model, long-term intake of a DON-containing diet significantly suppressed the growth of BALB/c mice and resulted in severe villus atrophy, structural disruption, and an altered villus height-to-crypt depth (V/C) ratio in intestinal segments such as the jejunum. The V/C ratio is a key indicator of intestinal absorptive function, and its deterioration reflects impaired nutrient absorption, which explains the significantly lower weight gain in DON-exposed mice compared to the control group. Importantly, both the culture supernatant and the degradation supernatant of *Lactobacillus rhamnosus* MY-1 significantly restored DON-induced reduction in IPEC-J2 cell viability and ameliorated ultrastructural damage. In mice, MY-1 effectively counteracted DON-induced growth retardation and markedly improved villus atrophy and structural injury in the jejunum and other intestinal segments. These results demonstrate that MY-1 can effectively mitigate the direct cytotoxic effects of DON and protect against its detrimental impacts on animal growth and intestinal morphology.

The integrity of the intestinal epithelium is highly susceptible to disruption by harmful factors within the luminal environment. In this context, oxidative stress and inflammatory responses triggered by exogenous toxins represent core mechanisms disrupting intestinal homeostasis (Maloy and Powrie, 2011; Zhu et al., 2023). Previous studies have confirmed that DON induces ROS generation, leading to redox imbalance and oxidative stress, which further activates pro-inflammatory signaling pathways, significantly upregulates pro-inflammatory cytokine expression, and initiates inflammatory responses (Mishra et al., 2014; Wang et al., 2019). Consistently, this study observed that DON exposure markedly increased MDA levels in IPEC-J2 cells, strongly upregulated mRNA expression of the pro-inflammatory cytokines TNF-*α* and IL-1α, and suppressed expression of the anti-inflammatory cytokine IL-4, confirming that DON simultaneously induces significant oxidative damage and inflammatory responses in intestinal epithelial cells. Current interventions against DON cytotoxicity primarily involve plant extracts or chemical substances such as chlorogenic acid, rosewood essential oil, and melatonin (Zhang et al., 2021; Xu et al., 2023; Chen et al., 2024). Although some studies indicate that *Lactobacillus rhamnosus* can alleviate DON-induced renal injury and anorexia or reduce toxicity via toxin transformation, these have largely focused on physiological observations at the animal level or protection of specific organs (Qu et al., 2019; Ma et al., 2022). However, the direct cellular and molecular mechanisms by which *Lactobacillus rhamnosus* alleviates DON induced oxidative stress and inflammation in the intestinal epithelium, the primary target of DON, are still unclear. This study demonstrates that treatment with the degradation supernatant of *Lactobacillus rhamnosus* MY-1 significantly ameliorates the cellular oxidative state, as reflected by reduced MDA content and enhanced T-AOC. Concurrently, a favorable shift in the inflammatory profile was observed, characterized by suppressed expression of the pro-inflammatory cytokines TNF-α and IL-1α and restored expression of the anti-inflammatory cytokine IL-4. This mechanism of coordinately mitigating oxidative stress and inflammatory imbalance aligns with the reported therapeutic mode of action of various natural bioactive compounds, such as polysaccharides (Hou et al., 2020). These findings provide new insights into the ability of *L. rhamnosus* MY-1 to alleviate DON-induced oxidative stress and inflammatory response at the cellular and molecular level, thereby contributing to the protection of intestinal epithelial cells. These protective effects could be attributed to a combination of DON detoxification and general probiotic activities, as the degradation supernatant has not been fully characterized.

Apoptosis, a key mechanism of programmed cell death, plays an important role in maintaining intestinal epithelial homeostasis and responding to toxic injury (Kang et al., 2019). In the present study, DON exposure significantly up-regulated the mRNA expression of the pro-apoptotic genes BAX and Caspase-3 in IPEC-J2 cells and promoted apoptosis. Consistent with these *in vitro* findings, DON also increased the expression levels of BAX and Caspase-3 in the duodenum and ileum of mice, accompanied by down-regulation of the anti-apoptotic gene BCL-2, suggesting the modulation of apoptosis-related gene expression in DON-induced intestinal injury. Treatment with *Lactobacillus rhamnosus* MY-1 effectively reversed these gene expression alterations and significantly reduced the expression of pro-apoptotic genes in both cellular and mouse models. These results demonstrate that *L. rhamnosus* MY-1 alleviates DON-induced apoptosis of intestinal epithelial cells by modulating the expression of apoptosis-related genes, thereby playing a key role in protecting the intestinal epithelial barrier. It should be noted that these observations are based on gene expression and morphological assessment; future studies quantifying apoptosis at the protein level would further substantiate these findings.

Tight junction proteins, as fundamental components of intestinal barrier function, play a critical role in maintaining intestinal homeostasis by regulating paracellular permeability (Ashida et al., 2011). Among them, occludin, ZO-1, and claudin-1 are core protein components that determine barrier integrity, and their expression levels directly influence barrier function (Grenier and Applegate, 2013; Jiang et al., 2021). This study found that DON exposure significantly suppressed the mRNA expression of ZO-1, Occludin, and Claudin-1 in IPEC-J2 cells. *Lactobacillus rhamnosus* MY-1 appeared to counteract this DON-induced downregulation, with notable increases in the mRNA levels of ZO-1 and Occludin. These in vitro observations were consistent with trends seen in animal experiments, where DON treatment was associated with reduced mRNA expression of tight junction proteins in a segment-specific manner: Claudin-1 expression was lower in the duodenum, while Occludin was decreased in the jejunum and ileum. Intervention with MY-1 tended to alleviate these changes, suggesting a possible supportive role in maintaining tight junction-related gene expression under DON challenge. The coordinated upregulation of tight junction protein mRNA levels across both models indicates that MY-1 may help preserve transcriptional programs related to barrier integrity. However, it is important to emphasize that these conclusions are based solely on mRNA measurements. Future studies incorporating protein-level techniques (e.g., Western blot or immunohistochemistry) are needed to directly evaluate tight junction protein abundance and localization, and to confirm functional barrier restoration.

The gut microbiota interacts with both the innate and adaptive immune systems of the host to maintain a dynamic equilibrium in the intestinal immune environment and control inflammatory responses (Yoo et al., 2020). Dysbiosis of the gut microbiota can promote inflammation and compromise epithelial barrier function by altering bacterial composition and metabolic activity (Al Bander et al., 2020). Previous studies have confirmed that deoxynivalenol significantly disrupts gut microbiota homeostasis, characterized by reduced alpha diversity indices (Chao, ACE, Shannon) and compositional shifts at both the phylum and genus levels, such as decreased abundance of beneficial bacteria (*Firmicutes*, *Actinobacteria*) and increased opportunistic pathogens (*Bacteroidetes*, *Proteobacteria*) (Zhai et al., 2022). Given that such microbial imbalance is a key aspect of DON-induced toxicity, it represents a potential target for nutritional intervention. Accordingly, functional dietary components, including polysaccharides from botanical sources like tea, jujube, and herbal formulas, have been shown to ameliorate dysbiosis by modulating bacterial composition (Ji et al., 2020; Huang et al., 2025; Tian et al., 2025). In the present study, DON exposure led to a marked reduction in alpha diversity indices (Chao1, Shannon, Simpson) and clear clustering separation in beta diversity analysis. We also observed significant enrichment of potentially harmful bacteria in DON-exposed mice, including members of the order Erysipelotrichales, family Erysipelotrichaceae, and genus *Dubosiella*. However, intervention with *Lactobacillus rhamnosus* MY-1 effectively counteracted DON-induced dysbiosis, not only restoring microbial alpha diversity but also promoting the proliferation of probiotic species (*Lactobacillus*). The specific microbial restructuring induced by MY-1—characterized by increased beneficial taxa (e.g., *Lachnospiraceae*, *Lactobacillus*) and decreased taxa often associated with dysbiosis (e.g., *Dubosiella*, *Erysipelotrichaceae*)—aligns with a microbiota composition that has been broadly linked to improved intestinal homeostasis in other studies (Ammar et al., 2023; Cui et al., 2023). Therefore, the alleviation of DON-induced intestinal injury observed in this study may be associated with this MY-1-mediated shift toward a more favorable gut microbial ecology. Its ability to selectively enrich beneficial bacterial communities while suppressing opportunistic pathogens underscores its potential in counteracting DON toxicity. Collectively, these results indicate that targeted modulation of the gut microbiota represents a promising strategy to alleviate DON-induced intestinal injury.

This study demonstrates the multifaceted protective role of *Lactobacillus rhamnosus* MY-1 against DON-induced intestinal injury via integrated mechanisms involving antioxidant activity, anti-inflammatory effects, apoptosis suppression, barrier restoration, and microbiota regulation. This systematic multi-pathway strategy underscores the value of the MY-1 probiotic strain in counteracting DON toxicity and lays an important theoretical foundation for developing comprehensive mycotoxin mitigation strategies based on microbial agents.

## 5 Conclusion

In summary, this study demonstrates that *Lactobacillus rhamnosus* MY-1 alleviates DON-induced intestinal toxicity through multiple mechanisms, including mitigation of oxidative stress and inflammation, modulation of apoptosis-related gene expression, restoration of tight junction-related gene expression, and improvement of microbial dysbiosis. The protective effects are likely supported by its DON degradation capability, although the chemical composition of the detoxified supernatant remains uncharacterized. The high degradation efficiency, combined with its dual detoxification strategy, clear safety profile, and systematic multi-target protective effects, distinguishes MY-1 from previously reported probiotic strains. Therefore, *L. rhamnosus* MY-1 represents a promising probiotic candidate for mitigating DON-induced intestinal damage, offering a viable and comprehensive strategy to counteract mycotoxin threats in animal production. Future studies will characterize the detoxified supernatant, and provide protein-level quantitative validation of apoptosis and tight junction integrity. Subsequently, the purified active components will be evaluated for their efficacy in large-scale animal models to facilitate industrial application.

## Data Availability

The data presented in the study are deposited in the NCBI Sequence Read Archive (SRA) repository, BioProject accession number PRJNA1417336.
