# Supplementary material for: Lactobacillus rhamnosus MY-1 alleviates deoxynivalenol-induced oxidative stress, inflammation, and gut microbiota dysbiosis both in vivo and in vitro
Source: Front Microbiol. 2026 Feb 16;17:1750402. doi: 10.3389/fmicb.2026.1750402 (PMC12950685; doi:10.3389/fmicb.2026.1750402)
Supplement: Supplementary file 1 [file Table_1.DOCX]

Suppl. Tab. 1 qPCR primer sequences for cellular and mouse samples.

| Sample Type | Target gene | Primer sequence (5'-3') |
| --- | --- | --- |
| Cellular | β-actin | F：CTGCGGCATCCACGAAACT |
|  |  | R：AGGGCCGTGATCTCCTTCTG |
|  | TNF-α | F：TTATCGGCCCCCAGAAGGAA |
|  |  | R：CGACGGGCTTATCTGAGGTT |
|  | IL-1α | F：TGCAATCATGACCACGCCCA |
|  |  | R：GCGACAAAGAGATGACTCGCI |
|  | IL-4 | F：ACTGATCCCAACCCTGGTCI |
|  |  | R：GCATCTGGAGAGATGGTGCC |
|  | BAX | F：GCCCTTTTGCTTCAGGGTTTC |
|  |  | R：CAATGCGCTTGAGACACTCG |
|  | BCL-2 | F：GATAACGGAGGCTGGGATGC |
|  |  | R：TTATGGCCCAGATAGGCACC |
|  | Caspase-3 | F：GGAATGGCATGTCGATCTGGT |
|  |  | R：ACTGTCCGTCTCAATCCCAC |
|  | ZO-1 | F：CCTGAGTTTGATAGTGGCGTTGA |
|  |  | R：AAATAGATTTCCTGCCCAATTCC |
|  | Occludin | F：ACCCAGCAACGACATA |
|  |  | R：TCACGATAACGAGCATA |
|  | Claudin-1 | F：ATTTCAGGTCTGGCTATCTTAGTTGC |
|  |  | R：AGGGCCTTGGTGTTGGGTAA |
| Mouse | GAPDH | F：GCAAATTCAACGGCACAGTCAAG |
|  |  | R：TCGCTCCTGGAAGATGGTGATG |
|  | ZO-1 | F：GCTTTAGCGAACAGAAGGAGC |
|  |  | R：TTCATTTTTCCGAGACTTCACCA |
|  | Occludin | F：TGAAAGTCCACCTCCTTACAGA |
|  |  | R：CCGGATAAAAAGAGTACGCTGG |
|  | Claudin-1 | F：TGCCCCAGTGGAAGATTTACT |
|  |  | R：CTTTGCGAAACGCAGGACAT |
|  | BAX | F：TTTAATGTCACGCACGATTTC |
|  |  | R：CCATGATGGTTCTGATCAGCTC |
|  | BCL-2 | F：TGAAGCGGTCCGGTGGATA |
|  |  | R：CAGCATTTGCAGAAGTCCTGTGA |
|  | Caspase-3 | F：AGAGACATTCATGGGCCTGAAATAC |
|  |  | R：CACCATGGCTTAGAATCACACACAC |
