# Supplementary material for: Lactobacillus rhamnosus MY-1 alleviates deoxynivalenol-induced oxidative stress, inflammation, and gut microbiota dysbiosis both in vivo and in vitro
Source: Front Microbiol. 2026 Feb 16;17:1750402. doi: 10.3389/fmicb.2026.1750402 (PMC12950685; doi:10.3389/fmicb.2026.1750402)
Supplement: Supplementary file 2 [file Table_2.DOCX]

Suppl. Tab. 2 Effects on growth performance of mice

|  | Parameters | Groups | | | |
| --- | --- | --- | --- | --- | --- |
|  |  | Control | MY-1 | DON | MY-1+DON |
| Male BALB/c mice | Initial average body weight (g) | 17.35±1.69 | 17.62±1.73 | 16.29±1.35 | 16.65±1.58 |
|  | Final average body weight (g) | 18.78±1.5 | 18.72±1.42 | 17.38±2.52 | 17.40±2.15 |
|  | Average daily feed intake (g) | 24.78±0.7 | 24.08±1.09 | 24.65±0.49 | 23.91±0.59 |
