# Supplementary material for: Lactobacillus rhamnosus MY-1 alleviates deoxynivalenol-induced oxidative stress, inflammation, and gut microbiota dysbiosis both in vivo and in vitro
Source: Front Microbiol. 2026 Feb 16;17:1750402. doi: 10.3389/fmicb.2026.1750402 (PMC12950685; doi:10.3389/fmicb.2026.1750402)
Supplement: Supplementary file 3 [file Table_3.DOCX]

Suppl. Tab. 3 Villi height, crypt depth and chorion-to-crypto ratio of small intestine

| Segment | Parameters | Control | | MY-1 | | DON | | MY-1+DON | |  |
| --- | --- | --- | --- | --- | --- | --- | --- | --- | --- | --- |
| Duodenum | Villus Height (μm) | 432.67±34.67 | | 444.00±36.00 | | 345.33±14.67** | | 411.33±21.33 # | |  |
|  | Crypt Depth (μm) | 139.00±9.00 | | 142.33±15.33 | | 147.67±12.67 | | 144.00±13.00 | |  |
|  | Villus Height/Crypt Depth (V/C) Ratio | 3.12 | | 3.14 | | 2.35** | | 2.86 # | |  |
| Jejunum | Villus Height (μm) | 444.00±29.00 | | 422.33±29.67 | | 320.00±27.00** | | 446.33±27.67 ## | |  |
|  | Crypt Depth (μm) | 132.00±8.00 | | 131.00±8.00 | | 163.33±24.67* | | 146.33±19.67 | |  |
|  | Villus Height/Crypt Depth (V/C) Ratio | 3.36 | | 3.23 | | 1.98** | | 3.09 ## | |  |
| Ileum | Villus Height (μm) | 227.00±8.00 | | 288.67±34.33** | | 208.33±15.67 | | 299.67±19.67** ## | |  |
|  | Crypt Depth (μm) | 138.67±15.67 | | 145.67±17.67 | | 176.67±16.67* | | 147.67±23.67 | |  |
|  | Villus Height/Crypt Depth (V/C) Ratio | | 1.65 | | 2.01 | | 1.19 | | 2.07 ## | |

Note: * indicates 0.01 < *P* < 0.05 and ** indicates *P* < 0.01 compared to control group; # denotes 0.01 < *P* < 0.05 and ## denotes *P* < 0.01 compared with DON group.
