# Supplementary figures and images for: Lactobacillus rhamnosus MY-1 alleviates deoxynivalenol-induced oxidative stress, inflammation, and gut microbiota dysbiosis both in vivo and in vitro
Source: Front Microbiol. 2026 Feb 16;17:1750402. doi: 10.3389/fmicb.2026.1750402 (PMC12950685; doi:10.3389/fmicb.2026.1750402)

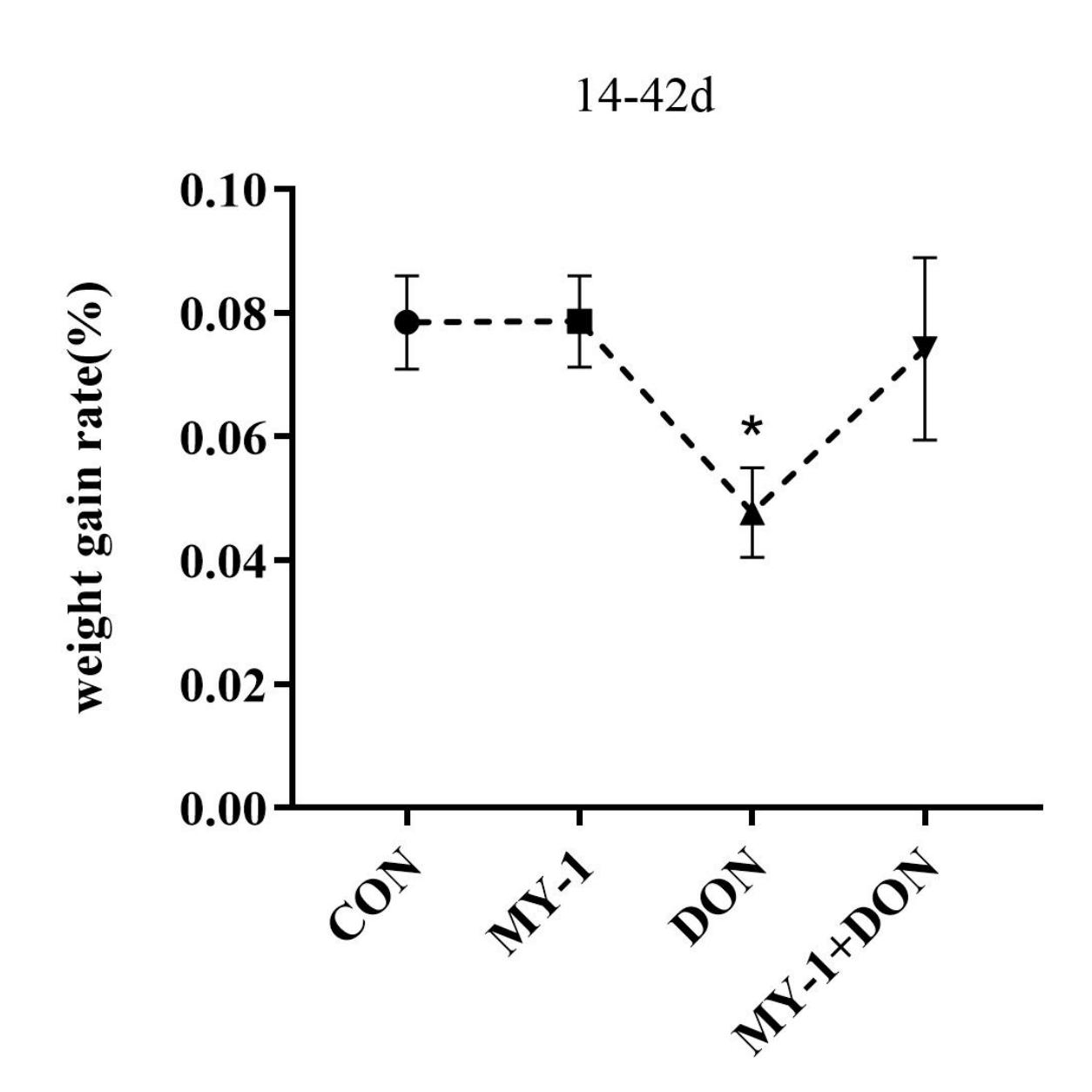

Supplement: Supplementary file 4 [file Image_1.JPEG]
